# An Exploration of Health Professionals' Views of Diet Intervention for Psychosis Management

**DOI:** 10.1111/inm.70073

**Published:** 2025-06-12

**Authors:** Kevin Williamson, John Baker, Nicola Clibbens

**Affiliations:** ^1^ School of Healthcare University of Leeds Leeds UK; ^2^ Centre for Nutrition and Behaviour, Grounded Research Hub RDaSH NHS Foundation Trust Doncaster UK; ^3^ Department of Nursing, Midwifery and Health Northumbria University Newcastle upon Tyne UK

**Keywords:** alternative therapy, dietary intervention, health professionals views, nutrition, psychosis management

## Abstract

Psychosis has a large impact on individuals and their families and current treatment approaches are not fully efficacious. The formation and function of the brain are dependent upon nutrients, supplied through the diet. Despite this, evidence indicates that the diets of people living with psychosis are nutritionally suboptimal. The aim of this qualitative research study was to seek the views of healthcare professionals experienced in psychosis management on the role and core components of dietary intervention for psychosis management. The study was conducted following the Medical Research Council's (MRC) framework's approach to the development and evaluation of complex healthcare interventions. The data were thematically analysed and constructed into four themes: (1) ‘*A desire for more knowledge on diet in relation to psychosis management*’; (2) *‘Balancing duty of care around diet within services’*; (3) *‘Health Professionals' perceptions of dietary habits of people with psychosis’*; and (4) ‘*Factors to consider when developing and implementing a diet intervention for psychosis*’. Findings from these key stakeholders suggest value for diet intervention within psychosis management, delivered by trained health professionals within National Health Service (NHS) mental health services. The evidence‐based diet intervention should be accessible to patients and should lead to the necessary dietary knowledge and skills acquisition for patients and their families. The principal recommendation following this research is to develop a diet intervention for psychosis management with additional stakeholder involvement, including NHS mental health service commissioners and academics responsible for health professionals' pre‐ and postregistration curricula.

## 1 Introduction

Diet and sound nutrition are important for all aspects of health, although little has been published on the views of professionals regarding the role of diet in the management of psychosis. Psychosis is one of the most life‐impacting conditions on quality of life and one of the most significant in terms of lifelong health outcomes and access to appropriate care (Ajnakina et al. 2021), despite its relatively low prevalence worldwide: 0.027% (95% CI: 0.022–0.032) (Jongsma et al. 2019). The onset of psychosis may have multiple contributory factors (Lieberman and First 2018; Kisely et al. 2024) and examples of the features of psychosis include changes in auditory and visual perceptions, alogia and anhedonia (Correll and Schooler 2020; Kirkpatrick et al. 2006). A combined approach of pharmacological and psychosocial intervention (PSI) is advocated in the United Kingdom by The National Institute for Health and Care Excellence (NICE 2022).

Pharmacological treatment is associated with metabolic side effects, such as dyslipidaemia and hyperglycaemia predisposing to diabetes, cardiovascular disease and weight gain (Pillinger et al. 2020). Additional side effects include suicidality, akathisia, sedation and cognitive impairment, all of which are hugely impactful on individuals, often leading to treatment avoidance (Stroup and Gray 2018). Furthermore, these medications do not work for everyone (Ramachandraiah et al. 2009), with rates of nonremission of symptoms following pharmacological treatment estimated at 20%–60% (Howes et al. 2022). Poor treatment response is multifactorial and linked to illness severity, impact of side effects and personal choice (Haddad et al. 2014). Likewise, nonengagement with PSI is estimated at around 13% (Villeneuve et al. 2010).

Best practice guidance in the UK cites a need to consider the broader health needs of individuals and their families and that early treatment, or better still, prevention is warranted (NICE 2022). Diet plays a fundamental role in the physiological, social and cultural aspects of the life of someone with psychosis, and as part of a holistic approach to treatment, has been regarded positively by nursing professionals (Prytys et al. 2011) and by patients (Jorm 2000). This may be due to the perceived lack of effectiveness of current treatment strategies or because diet could represent an aspect of treatment that provides an individual with a sense of autonomy.

## 2 Background

Diet quality affects brain structure and function (Wachs 2000). Nutrients, such as the amino acid tyrosine, are essential for dopamine synthesis and neurotransmission (Wykes et al. 1971), which is altered in those with psychosis (Kesby et al. 2018). There are a range of nutrients known to positively impact brain cell health, including omega‐3 fatty acids (Simonelli‐Munoz et al. 2012), vitamins B6, B9, and B12 and vitamins C, D and E, which are all known to be deficient in those with psychosis (Firth et al. 2018). There is evidence through meta‐analysis of randomised controlled trials (RCTs) of improvement in psychosis symptoms following nutrient supplementation with several nutrients, including omega‐3 fatty acids and B vitamins (Firth et al. 2017), although most of these trials do not address potential issues with the acceptability of long‐term diet supplement usage in those experiencing psychosis versus whole food or overall dietary change strategies.

People with psychosis consume diets that do not support a healthy brain. A cohort of patients experiencing psychosis (*n* = 88) was found to consume diets low in beneficial vitamins and minerals, yet significantly higher in caffeine compared with general population averages of 471.6 versus 254.2 mg (471.6 ± 584.6 vs. 254.2 ± 384.9 mg, *t* = 6.664, *p* < 0.001) (Strassnig et al. 2006). A recent study using self‐reported diet data found lower than recommended consumption of fruit and vegetables, with 53% consuming ⩾ 3 fruit servings per week and 58% consuming ⩾ 3 vegetable servings per week (Martland et al. 2023). Likewise, the study reported expected omega‐3 fatty acid deficiency in those surveyed due to a large proportion not eating fish (63.1%), which is the primary source of omega‐3 fatty acids (Simonelli‐Munoz et al. 2012). Diet data were collected from a cohort of individuals with first‐episode psychosis (*n* = 143), via completion of 4‐day food diaries, to produce mean nutrient intakes that were then compared with the UK population average (Williamson et al. 2015). The findings included a higher intake of processed foods and processed sugar compared with the UK average despite a similar total energy intake (Williamson et al. 2015). Likewise, several of the micronutrients were lower than the national average; most notably selenium, with over 50% of participants not meeting the lower reference nutrient intake (Williamson et al. 2015).

There are known links between foods and nutrients and psychotropic medication. Olanzapine was a predictor of added sugar consumption from sugar‐sweetened beverages in those given a diagnosis of schizophrenia (Lambert et al. 2024). This may pose a health risk through the impact of excess energy leading to weight gain, which may be exacerbated by conditions, such as psychogenic polydipsia, which is known to affect 6%–20% of those with psychosis (Kirino et al. 2020). Caffeine is known to act on dopamine receptors, leading to an increased requirement for antipsychotic medication in those with psychosis (Broderick et al. 2005).

The role of dietary intervention as part of psychosis management has been advocated within best practice for mental health professionals for almost two decades (Department of Health 2005) and as a direct recommendation to the United Kingdom's Department of Health and Social Care (DHSC) following a cross‐Parliamentary inquiry (Associate Parliamentary Food and Health Forum 2008). Meeting a patient's dietary needs forms a fundamental part of the mandate of care across nations, with an example being the United Kingdom's National Health Service (NHS) Constitution, which indicates that all patients must be able to access sound nutrition as part of their healthcare journey (NHS Constitution for England, DHSC 2021). Despite this, an evidence‐based diet intervention is not offered as part of routine care for psychosis management. The aim of this study therefore was to explore health professional stakeholders' views on the role and core components of providing diet intervention as part of psychosis management.

## 3 Methods

### 3.1 Study Design

An inductive approach was taken with this qualitative study, which was rooted in Pragmatism and was aligned with the Medical Research Council's (MRC) framework for the development and evaluation of complex healthcare interventions (Skivington et al. 2021). The MRC Framework recommends that the first step in developing a new complex healthcare intervention is to systematically review published literature on the topic and if the evidence does not support an existing intervention, then one should be developed (Skivington et al. 2021). They recommend that the next step required to develop a new intervention is to seek the views of key stakeholders on the topic. This study was therefore conducted following a systematic review and meta‐analyses of published RCT evidence of diet interventions for psychosis management, which provided no evidence of an effective intervention (unpublished). The findings of this study are reported in line with the COREQ checklist (Tong et al. 2007).

### 3.2 Study Setting

Data collection was conducted with a sample of health professionals employed within a mental health and community NHS Trust in England, which spans two counties and comprises urban and rural populations. The Trust is commissioned to deliver inpatient and community services that include psychosis management, such as early intervention in psychosis and community mental health services.

### 3.3 Ethical Consideration

The study was sponsored by the University of Leeds. The study protocol, participant information sheet (PIS) and study topic guide were reviewed by four NHS research volunteers (two retired mental health nurses and two individuals with lived experience of psychosis) via email communication prior to submission for ethical review. Some minor amendments were made to the study protocol following their review, which they believed would add clarity to the rationale for conducting the study. Favourable opinion from the National Research Ethics Committee (NREC) and the Health Research Authority (HRA) (IRAS: 261606) was obtained following permission to conduct the study being granted by the NHS Trust.

### 3.4 Recruitment

The sample was purposively recruited through direct email communication to potential participants and via attendance (K.W.) at team meetings. Participants were professionals with experience in the last 2 years of providing care and treatment for psychosis in inpatient or community NHS mental health services. Eligibility to participate in the study was defined as professionals who held a current employment contract within the NHS Trust and their availability to attend one of the series of focus group sessions.

### 3.5 Data Collection

Potential participants were asked to provide and sign a form denoting their informed consent. Data collection was led by a researcher/Registered Nutritionist with prior experience in developing and delivering diet intervention as part of psychosis management (K.W.; PhD, MMedSci). Participants were invited to share their professional occupations at the time of data collection, which was recorded by the researcher (K.W.). Data collection strategies were discussed and agreed upon with two registered mental health nurses (J.B. and N.C.; both PhD). Focus group interview method was chosen to gain value through interaction and discussion between participants (Kitzinger 1995). The total sample was determined by mutual agreement between all three researchers and followed the recommendations of Guest et al. (2017) who suggested that over 90% of all themes on a particular topic would be discovered through conducting between three and six group interview sessions. The topic guide utilised to aid data collection was developed by KW and refined by all researchers and involved from those with lived experience, following a review of the literature. The guide included several open questions designed to elicit knowledge and experience of the role of diet for brain health and for psychosis management. Example questions included ‘Would you please describe what you know about the effect of diet on the brain’ and ‘is there anything else you feel important to consider regarding diet and nutrition in the management of psychosis’. All focus group sessions were conducted face‐to‐face in NHS premises between 14 November 2019 and 6 February 2020 with only one researcher (K.W.) and participants present during data collection. The sessions were held in premises that were familiar and convenient for the participants and refreshments, including water, tea and coffee were provided on arrival to help put participants at ease. Prior to the start of each group session, participants were asked to speak openly and honestly about their experiences. Each group session had a minimum of two and a maximum of eight participants (Kitzinger 1995), with attendance slots booked in advance. Each participant was given a numerical identifier to help manage individual contributions during the event, as they were asked to hold it during active discussions when they wished to speak. The facilitator's (K.W.) role included managing these contributions, although conversations occurred effortlessly, and it was not required. All focus group sessions were audio‐recorded and transcribed. No additional field notes were taken, but the researcher kept a reflective diary throughout the conduct of the study to support the analysis and report writing phases. Two researchers (K.W. and N.C.) knew some, but not all, of the research participants prior to the study and participants were made aware of the researcher's (K.W.) interest in and aims for conducting the study.

### 3.6 Analysis

Following data transcription, thematic analysis was conducted following Braun and Clarke's (2006) six‐step approach. All researchers completed Step 1, data familiarisation prior to coding to help the subsequent generation of themes, and Steps 2–5, which were the generation of initial codes through to Step 5 defining and naming themes were led by K.W. with guidance from J.B. and N.C. Step 6, production of the report, was led by K.W. with contributions from J.B. and N.C. Themes were generated iteratively from the data through the development and refinement of a series of coding trees, which diagrammatically represented groups and subgroups of codes and their linkage to the theme and each other. Versions of the coding tree were developed using a variety of methods, including use of Microsoft Excel, Microsoft Word and pen and paper. Data handling was managed through NVivo software (Version 12; QSR International Pty Ltd.). Due to the unique nature of several participants' roles and occupations, a decision was made by the researchers not to report data by individual profession as this could breach the anonymity of participants.

### 3.7 Enhancing Trustworthiness

The approach developed by Nowell et al. (2017) was followed to ensure trustworthiness of the findings, including use of reflexive journalling and the development of an audit trail throughout the cyclical process of theme generation to aid the recursive process of theme generation and allow full transparency throughout the process. All researchers independently reviewed codes, categories and themes, and each step of the process was discussed by all researchers as a team.

## 4 Findings

### 4.1 Sample Characteristics

The five focus groups comprised 26 participants (*n* = 2; 3; 7; 8; 6) and spanned the professional groups of nursing (*n* = 15), psychiatry (*n* = 2), allied health professions comprising psychology, occupational therapy and social work (*n* = 5) and support workers (*n* = 4). Of the 26 participants, 19 worked in community services and seven in inpatient services, summarised in Table 1. The focus group sessions ranged between 15 and 47 min in duration. No participant declined to participate or withdrew their consent during the study.

**TABLE 1:** Participant characteristics represented by focus group session.

Group ID	Geographical area	Clinical setting	Professional group representation	Duration (minutes)
A	Mixed	Mixed	Nursing (1); AHP (1)	15
B	1	Mixed	Nursing (1); AHP (1); support worker (1)	35
C	3	Community	Nursing (4); AHP (1); support worker (2)	22
D	2	Community	Nursing (3); AHP (2); support worker (1); psychiatry (2)	30
E	2	Inpatient	Nursing (6)	47

#### 4.1.1 Themes

The thematic analysis resulted in four themes: (1) ‘A desire for more knowledge on diet in relation to psychosis management’; (2) ‘Balancing duty of care around diet within services’; (3) ‘Professionals' perceptions of dietary habits of people with psychosis’; and (4) ‘Factors to consider when developing and implementing a diet intervention for psychosis’.

##### 4.1.1.1 Theme 1: A Desire for More Knowledge on Diet in Relation to Psychosis Management

Participants expressed a desire for more knowledge on dietary intervention for psychosis management due to its perceived value as a holistic treatment option. The participants believed that a dietary intervention had the potential to empower patients through a self‐management strategy, which could improve their physical health because changes in their diet have the potential to reduce the need for medication if used as an adjunct to existing treatments.

‘If there are alternatives to try, to alleviate some symptoms, because some of the medications can be horrendous, can't they?… if there's something that can complement the medication then why not look at it’. (Group A)

Participants reported a lack of appropriate knowledge of diet and nutrition, citing private study (Group B), anecdotal information from the internet, from those experiencing psychosis (Group C) or from one or two sessions on patient feeding during preregistration professional training, as the basis for their current knowledge (Group D). They valued evidence‐based practice and thus acknowledged the weakness of relying on informal sources such as the Internet or information gleaned from patients (Group C). One participant had failed to access postregistration nutrition training (Group C), whilst another had been discouraged from studying nutrition for their nursing degree thesis because ‘*nutrition is not a nursey subject*’ (Group B).

Participants suggested that a diet intervention for psychosis management should be incorporated as part of dietetic, nutritionist, nursing and other health professional curricula (Group D). Irrespective of the provision of adequate training, there was debate as to which professions were best placed to advise on diet. Some participants believed that a basic grasp of nutrition concepts for psychosis management would help all health professionals, whereas others believed that diet and nutrition was a specialism and therefore the responsibility should sit with nutrition or dietetic professionals, who had the time and requisite expertise to deliver it.

‘It would be good [to have nutrition training], but I think it would never replace having access to a nutritionist’. (Group D)

Participants linked their current lack of knowledge to their fear of providing misinformation about diet and nutrition to patients. They equated this with professional negligence.

‘If you've got people with diabetes or on other medications where their diet can make them be unwell, you don't want give the wrong advice, do you? We just can't’. (Group D)

To overcome these fears, participants suggested that alongside incorporating nutrition and diet into health professionals' curricula, there must also be a mechanism developed and implemented in practice to monitor knowledge acquisition. Activating this mechanism could provide assurance both to organisations and to health professionals that they have sustained competence to deliver a diet and nutrition intervention safely and effectively.

##### 4.1.1.2 Theme 2: Balancing Duty of Care Around Diet Within Services

Participants believed that health services and health professionals have a duty of care to support patients' dietary needs. Participants were frustrated that nutrition and diet, a fundamental part of health and wellbeing, are not mandated as part of care delivery. One example shared by participants was inadequate assessment of diet through the annual health check for those with psychosis, with only ‘… *tick a box*’ options (Group A and Group C), which they believe leads to apathy from patients (Group C).

Participants believed that more should be done by mental health services to support those with psychosis to have optimum nutritional status, such as using diet supplementation, where individuals were deficient. Further to this, participants wanted commissioners and NHS Trusts to ensure the amount and nutritional quality of the food served on NHS mental health wards was commensurate with the health needs of those living with psychosis. This was particularly frustrating for participants because they believed that health services and healthcare professionals should be promoting and modelling healthy behaviours, but the current food provision often undermined this.

‘On the ward… pudding and custard… it's sort of the staple pudding that's served for most meals… If we're trying to educate them into how the diet can help and support with symptoms, then maybe that should start in the hospitals’. (Group B)

Participants reflected that some who experienced psychosis were sad after they were discharged from the ward because they gained weight while there. The participants suggested that the food provided by the service had contributed to the individuals' weight gain, whilst also possibly engendering these unhealthy dietary habits within patients' postdischarge. Health professionals expressed guilt that they may not have fully upheld their duty of care.

‘They come out, and then by the time I see them they're like, oh I can't believe that I've put on this much weight. But it's because they've been on the ward for however long and they've got into that routine’. (Group B)

A further tension for health professionals was their concern about the impact on therapeutic relationships if they pushed healthy dietary behaviours too much ‘sounding like a broken record and they're just telling you to go away’. (Group A). This was compounded by some familial carers who they believed reinforced bad dietary habits.

##### 4.1.1.3 Theme 3: Professionals' Perceptions of Dietary Habits of People With Psychosis

The data suggested that professionals believe that people with psychosis are different to the general population in terms of their dietary habits. Partly a reflection of psychosis symptoms leading to distracted thoughts and a lack of sufficient concentration to buy food and prepare meals, and partly through the impact of medication. The data also revealed the presence of negative judgements and a lack of optimism from some participants regarding the characteristics and behaviour of people experiencing psychosis.

‘I think, perhaps, the demographic that traditionally people with psychosis… there are always elements of poor diet and poor nutrition. … It's, I do think the demographic is probably, you know, the impacts are from a socio‐economic status, with the resources that are available to them, as well’. (Group A)

Participants also believed that patients ‘traditionally prefer processed food’ (Group B) and in dietary terms are *‘… in need of a quick fix*’ (Group B). One explanation for these food choices was that having psychosis leads to craving certain foods as a source of pleasure or comfort, particularly while unwell (Group D). Participants perceived that these foods acted as a proxy for ‘pleasure’ to balance out the unpleasant experience of psychosis, indicating too that health and care needs were not prioritised.

‘… maybe looking at food as a source of pleasure, so when you've got a lot of stuff going on that's displeasurable, but maybe the chocolate and the sweets and the crisps that offer the pleasure, maybe that's what they put importance on and getting some pleasure back in life rather than thinking, I need to eat a varied diet’. (Group B)

Participants described psychosis‐associated reduction in motivation impeding patients' ability to engage in dietary support from health professionals (Group A). This was expressed as frustration at ‘… the missed opportunity’ this represented (Group B). These views were balanced with compassion for the personal barriers people with psychosis face, particularly regarding their struggle to access nutritious foods, due to limited income or access to appropriate support and guidance around healthier dietary choices (Group B and Group D).

##### 4.1.1.4 Theme 4: Factors to Consider When Developing and Implementing a Diet Intervention for Psychosis

Participants believed dietary intervention within psychosis management should start at the earliest opportunity. One example was in the acute mental health wards (Group B; Group C) because initiating diet intervention could parallel other treatments, like medication (Group C). Ward environments would provide patients time and access to health professionals to develop new dietary habits and instil a sense of autonomy. Participants believed that patients on the ward would also be receptive to a diet intervention because they often seek activities to engage in whilst there (Group B). One participant suggested a potential benefit to initiating diet intervention on the ward is that it would then be viewed ‘as a normal part of their treatment’ (Group C). One challenge was that those with psychosis may lose their newly acquired dietary skills postdischarge through a lack of knowledge consolidation and lost contact with key professionals (Group B).

‘I'd be really keen to do some sort of sessions with people, maybe in a group, but when people come in they're not well and then when they are well they're not here long usually until they go back into the community’. (Group E)

Participants believed that diet interventions should have realistic outcomes, accessible to all who experience psychosis, including those in the community. Participants felt that having such outcomes would allow monitoring and evaluation of dietary changes at different time points, where improvements could act as further motivational aids for individuals. Participants also suggested that being able to share outcome data with an individual who highlighted improvement could help strengthen the therapeutic relationship, which they believed was key to successful intervention. Participants believed diet intervention should include core skills acquisition like meal preparation (Group B) because these skills may have been lost (Group E).

‘Like saying to someone cut down on your takeaways… to one day a week to begin with and eat some healthier meals. It doesn't have to be fancy cuisine… just better convenience foods maybe’. (Group B)

Participants believed that the option for one‐to‐one or group delivery of sessions, including skills‐based sessions would be beneficial, allowing some ‘learning from each other’ (Group D). Likewise, the location of sessions should be accessible to those experiencing psychosis (Group D). Participants identified that a diet intervention should also be able to support people living in unstable housing, with limited budgets or access to cooking equipment (Group C and Group D).

Accessibility of the diet intervention by patients outside standard service hours was something participants deemed important, with options ranging from an out‐of‐hours service to handouts, a website or an app (Group A, Group B, Group D and Group E). These health professionals described the advantages of technology as 24‐h accessibility to individuals when normal face‐to‐face support isn't provided, particularly in the community (Group C) and their interactivity, allowing individuals to track their progress, thus negating memory issues.

‘People do like apps… having that there when that person's gone, so you can check back in… they do make you a bit more accountable… It reminds you’. (Group D)

A blend of face‐to‐face service provision and out‐of‐hours digitally focussed diet intervention may best meet individuals' needs (Group D).

## 5 Discussion

This study explored the views of health professionals on the role of diet in psychosis management. The data within theme one indicated a positive response from participants to the concept of diet intervention within psychosis management. The need for appropriate health professional training and subsequent knowledge and competency assessment was deemed essential for the intervention to be delivered safely and effectively. This fits with nurses' professionalism in terms of seeking out safe, evidence‐based practice and concurs with a previous study where nurses and other health professionals working in mental health services were keen to receive nutrition education to better care for individuals they cared for (Clibbens and Williamson 2016). There was a lack of clarity about whom was best placed to provide a diet intervention, with some believing that it formed a part of their role as nurses or allied health professionals, whereas others suggested that this required nutrition and dietetic professionals' input. With appropriate training, dietary intervention could be part of advanced practice for mental health nurses and sit alongside popular specialisms such as psychotherapy or prescribing (Hurley et al. 2022). Irrespective of whether a diet intervention becomes the responsibility of one profession or several, it is clear that training will be required, and this will require buy‐in from academic institutions that deliver health professions curricula. Likewise, it will also require buy‐in from psychosis treatment service providers in terms of supporting the concept and then agreeing for professionals to have time to undertake the training and implement this in practice (Mathers et al. 2012).

The data indicated that whilst a formal dietary intervention for psychosis management is not currently provided, there are dietary components in existing practice, such as the provision of meals in inpatient wards. Participants raised a strong internal conflict because they believed that the current standard of care around diet was suboptimal, and they felt powerless to change that in their current practice. They felt they lacked the knowledge and support to challenge incidences of poor dietary care for those experiencing psychosis. The data suggested that health policy should therefore reflect the benefit and importance of diet intervention for psychosis management to help embed this more robustly and consistently. Participants recommended mandating dietary intervention to make it clearer to those with psychosis and their families that this was important for their care and help services provide a more consistent approach to diet intervention. They believed this would help build and protect the therapeutic relationship that they had with those who experience psychosis, which is critical to successful psychosis management (Crits‐Christoph et al. 2019).

The data within theme three could be perceived as a sense of negative judgement and stigma on those with psychosis, with examples such as ‘*the demographic that traditionally people with psychosis’*. Nordt et al. (2006) reported that psychiatrists, psychologists and nurses exhibited negative stereotypes towards those who are mentally unwell with similar frequency to the general population. There was, however, a sense of compassion from participants that any unhealthy behaviour should actually be excused because of the impact of psychosis on individuals. There is also the potential that the belief that those with psychosis have different dietary habits stems from a lack of participants' knowledge of dietary habits, as indicated by the data in theme one. Many of the examples cited by participants, such as increased use of takeaways and foods that provide comfort are equally true of the general population (Weaver et al. 2014). The sense of negative judgement expressed by some participants may represent frustrations following a lack of awareness when an individual is ready to make behaviour changes or the misconception that behaviour change is ‘common sense’ Kelly and Barker (2016). Irrespective of the root of biases or potential prejudices, this is worthy of exploration during the development of a diet intervention for psychosis management.

The data in theme four advances this research by providing health professionals' views on the key features of diet intervention for psychosis management. These include the need to begin early in and be accessible throughout, the psychosis care pathway. This ties in with the wider models for psychosis management, which have long advocated early intervention (Birchwood et al. 1998). Accessible intervention throughout the care journey was deemed essential, with the use of technology being advocated to extend the hours of service and facilitate individual self‐management and accountability. This parallels with wider literature and discussion on the topic citing that digital applications are in widespread usage in healthcare settings (Ventola 2014), however, a review of their efficacy at improving health outcomes, comprising 172 RCTs concluded that the evidence was weak and warranted further research with improved methodologies (Iribarren et al. 2021). Participants considered the concept of a diet intervention being tailored to the individual's goals and circumstances and linked to their readiness to change to be important and may act as a means to improve memory retention and uptake. The concept of tailoring diet interventions to support individual skills and knowledge acquisition has been seen as a critical aspect of successful implementation (De Roos and Brennan 2017), and as a means to improve knowledge retention (Weiss et al. 2003).

### 5.1 Strengths and Limitations

A strength of this study was that it is the first to explore the value of diet intervention as a part of psychosis management and, as such, it has provided key data that would help with (a) the development and (b) the successful implementation of such an intervention for those with psychosis. A further strength was seeking the views of those with lived experience of psychosis care on study documentation. Future studies could be improved by the involvement of people with lived experience throughout study delivery. Further to this, future research should conduct a similar study exploring the views of those with lived experience of psychosis. One of the main limitations when seeking the views of health professionals was participants' general lack of awareness of the topic, which may account for the relatively short duration of some of the focus group sessions (see Table 1) and a lack of divergence in the data. If participants had been provided with greater knowledge prior to the focus group session, it may have resulted in additional perspectives. A second limitation was the decision against member checking of the focus group transcripts. There was the danger that participants may have altered their views following participation; however, that may, in fact, have added to the study findings. A third limitation was that the professionals were all employed within the same NHS Trust. Health professionals working within a different Trust with different approaches and policies may have provided richer perspectives on the data.

## 6 Conclusion

Health professionals' views suggest merit in dietary intervention for psychosis management; however, an increase in professional knowledge via evidence‐based training and organisation‐wide buy‐in would be required ahead of interventional delivery within services. Diet intervention would require honest and transparent conversations as part of routine care delivery and should include familial carers as well as patients.

## 7 Relevance to Practice

There is a role for mental health nurses in the delivery of a diet intervention as part of psychosis management, and they would be well placed to help tailor diet intervention and support for individuals to support effective behaviour change. This must, however, be underpinned by appropriate training and subsequent monitoring of those professionals to ensure safe practice. Likewise, nurses and other health professionals must find the balance between maintaining a therapeutic relationship and their duty of candour with respect to difficult conversations around dietary habits and an individual's need for dietary change.

## Author Contributions

K.W. led the study design, delivery and regulatory and ethical approvals process, with support from J.B. and N.C. K.W. led participant recruitment, data collection and analysis, and K.W. led the manuscript development with significant contributions from J.B. and N.C. All authors are in agreement with the submitted version of this manuscript.

## Conflicts of Interest

The authors declare no conflicts of interest.

## Data Availability

The data that support the findings of this study are available on request from the corresponding author. The data are not publicly available due to privacy or ethical restrictions.
